# Point-of-care 3D printing: a low-cost approach to teaching carotid artery stenting

**DOI:** 10.1186/s41205-021-00119-3

**Published:** 2021-09-02

**Authors:** Pieter De Backer, Charlotte Allaeys, Charlotte Debbaut, Roel Beelen

**Affiliations:** 1 IBiTech-bioMMeda, Ghent University, Ghent, Belgium; 2 Orsi Academy, Melle, Belgium; 3 Faculty of Medicine and Health Sciences, Ghent University, Ghent, Belgium; 4 OLV Hospitals Aalst-Asse-Ninove, Ghent, Belgium

**Keywords:** Carotid Artery Stenting, 3D Printing, Training, Surgical Education, Phantom

## Abstract

**Background:**

Carotid Artery Stenting (CAS) is increasingly being used in selected patients as a minimal invasive approach to carotid endarterectomy. Despite the long standing tradition of endovascular treatments, visual feedback during stent-deployment is impossible to obtain as deployment is performed under fluoroscopic imaging. Furthermore, the concept of stent-placement is often still unclear to patients. 3D Printing allows to replicate patient-specific anatomies and deploy stents inside them to simulate procedures. As such these models are being used for endovascular training as well as patient education.

**Purpose:**

To our knowledge, this study reports the first use of a low-cost patient-specific 3D printed model for teaching CAS deployment under direct visualization, without fluoroscopy.

**Methodology:**

A CT-angiogram was segmented and converted to STL format using Mimics inPrint™ software. The carotid arteries were bilaterally truncated to fit the whole model on a Formlabs 2 printer without omitting the internal vessel diameter. Next, this model was offset using a 1 mm margin. A ridge was modelled on the original vessel anatomy which was subsequently subtracted from the offset model in order to obtain a deroofed 3D model. All vessels were truncated to facilitate post-processing, flow and guide wire placement.

**Results:**

Carotid artery stents were successfully deployed inside the vessel. The deroofing allows for clear visualization of the bottlenecks and characteristics of CAS deployment and positioning, including stent foreshortening, tapering and recoil. This low-cost 3D model provides visual insights in stent deployment and positioning, and can allow for patient-specific procedure planning.

**Conclusions:**

The presented approach demonstrates the use of low-cost 3D Printed CAS models in teaching complex stent behavior as observed during deployment. Two main findings are illustrated. On one hand, the feasibility of low-cost in-hospital model production is shown. On the other hand, the teaching of CAS deployment bottlenecks at the carotid level without the need for fluoroscopic guidance, is illustrated. The observed stent characteristics as shown during deployment are difficult to assess in radiologic models. Furthermore, printing patient-specific 3D models preoperatively could possibly assist in accurate patient selection, preoperative planning, case-specific training and patient education.

**Supplementary Information:**

The online version contains supplementary material available at 10.1186/s41205-021-00119-3.

## Introduction

### Background

Carotid artery stenosis affects 10 % of the population by the age of 80, and is associated with an increased risk of cerebrovascular events with major consequences on physical and neurological level [1–3]. Worldwide, carotid revascularization is advised for stroke prevention in moderate or severe carotid stenosis [4]. The revascularization can be performed by carotid endarterectomy (CEA) or Carotid Artery Stenting (CAS) [5, 6]. CEA, i.e. removal of the atherosclerotic plaque via a neck incision, has been the standard treatment since the early fifties [1, 7–10]. The introduction of endovascular carotid techniques 3 decades ago [11] demarked a shift towards the less invasive CAS approach [7, 9].

CAS is shown to be a safe long term alternative to CEA in symptomatic carotid stenosis for patients under 70 years, provided that both CEA and CAS are technically feasible [12]. CAS is associated with a slightly lower risk for periprocedural myocardial infarction when compared to CEA [6]. However, CAS is associated with a higher risk of stroke or death within 30 days of treatment, mostly attributable to an increase in periprocedural stroke in patients older than 70 years.

Today’s vascular surgery shifts towards minimal invasive techniques because of advantages such as shorter recovery periods, decreased postoperative pain and discomfort, smaller incisions, and shorter in-hospital stays [8, 9]. Particularly for CAS, a shorter recovery period, less procedural discomfort and an enhanced physical function in the first post-operative year is observed [9, 13]. CAS is preferred over CEA in specific indications including prior history of radiotherapy or neck dissection, contralateral laryngeal nerve damage, contralateral carotid occlusion, re-stenosis after CEA, and in case of high risk for surgery due to comorbidities [2, 3, 6, 9]. A more recent approach to transfemoral CAS is a transradial access. Even newer is the transcarotid artery revascularization (TCAR). In TCAR, the carotid artery is directly accessed endovascularly through a small incision in the neck and flow reversal is applied to avoid procedural distal embolization, which is one of the main concerns in CAS. Thus, TCAR can also avoid unfavorable aortic arch anatomy which can be a bottleneck in transfemoral CAS.

### Patient selection and preoperative planning

CT-angiogram (CTA) is often considered the gold standard in non-invasive carotid imaging and diagnosis [14]. Although new techniques allow for enhanced virtual 3D projections on a 2D computer display, these are not always readily available. Interpretation and visualization of complex cases is aided by converting the CTA to a 3D printed model [15]. 3D models facilitate the geometrical and spatial understanding of the arterial tree, especially in case of anatomical variants, high tortuosity and abnormalities of the carotid artery [16]. Ideally, a patient-specific 3D model could enhance the patient selection and the pre-operative planning by enhancing the surgeon’s understanding of the patient-specific 3D anatomy [17, 18].

The patient’s understanding on the other side, is often very limited due to the technical nature of CAS, and a lack of medical background. As such, patients do often not entirely comprehend the procedure they will undergo and can hardly estimate the involved risks [16]. Informing the patient based on his/her 3D model instead of two-dimensional (2D) imaging, as well as adding tactile 3D information to the planar CT imaging, leads to better patient education and enhance informed consent [16, 17].

### Training of CAS

To mitigate the increased stroke risk in CAS, it thus seems imperative to further optimize this endovascular technique through training, proper patient selection and pre-operative planning [7]. Outcomes in CAS are shown to be heavily dependent on operator training and skill [19] and CAS attempts by inexperienced surgeons lead to predictable poor results [20]. As such, the hospital and surgeon CAS volume is a major determining factor in the clinical outcome [7, 21].

Little training and poor technical skills lead to higher complication rates, higher fluoroscopy, increased nephrotoxic contrast volume usage, and longer procedural durations [1, 9]. Hence, besides being fully credential in peripheral endovascular techniques, hands-on training is necessary to enhance clinical proficiency for this specific endovascular procedure [1, 5, 9]. During carotid stenting teaching, awareness needs to be created on procedure specific features such as stent foreshortening, recapping, tapering, recoil and jumping of the proximal stent end. Dual layer carotid stents can shorten up to 28 % in length during deployment [22], which is crucial for correct positioning. This final positioning can be difficult to objectivate or estimate on 2D fluoroscopy for trainees. Recoil and jumping of the stent are very brief events and as such these might also be less easily objectivated during fluoroscopy.

Nowadays, training is based on endovascular simulation modules and industry-sponsored courses [1, 5, 9, 23]. These courses can provide a shortened learning curve, address specific features and hence may limit complications [1, 7]. Planning and practicing on 3D printed patient-specific models preoperatively can as such facilitate a straightforward approach by reducing X-ray exposure reduce and operation time [24]. To our knowledge, in hospital training is rarely done due to lacking infrastructure and lacking knowledge in the fabrication of cost-efficient models.

Due the increased use of 3D Printing in healthcare, a shift towards in-hospital 3D Printing services is taking place globally. More hospitals start to insource 3D Printing, driven by the increased printer performance, lower production costs, shorter time-to-product and easy-to-use 3D editing programs. A particular use case of this so-called Point-Of-Care printing is the pretreatment printing of vascular anatomy in support of endovascular procedures.

The current research trend focusses on building simulations for fluoroscopic evaluation but no work has been done in visualising CAS bottlenecks with the naked eye, without fluoroscopy. In this paper, we present a cost-effective workflow to perform CAS on a patient-specific 3D model without the use of fluoroscopy.

## Methodology

**Fig. 1:**
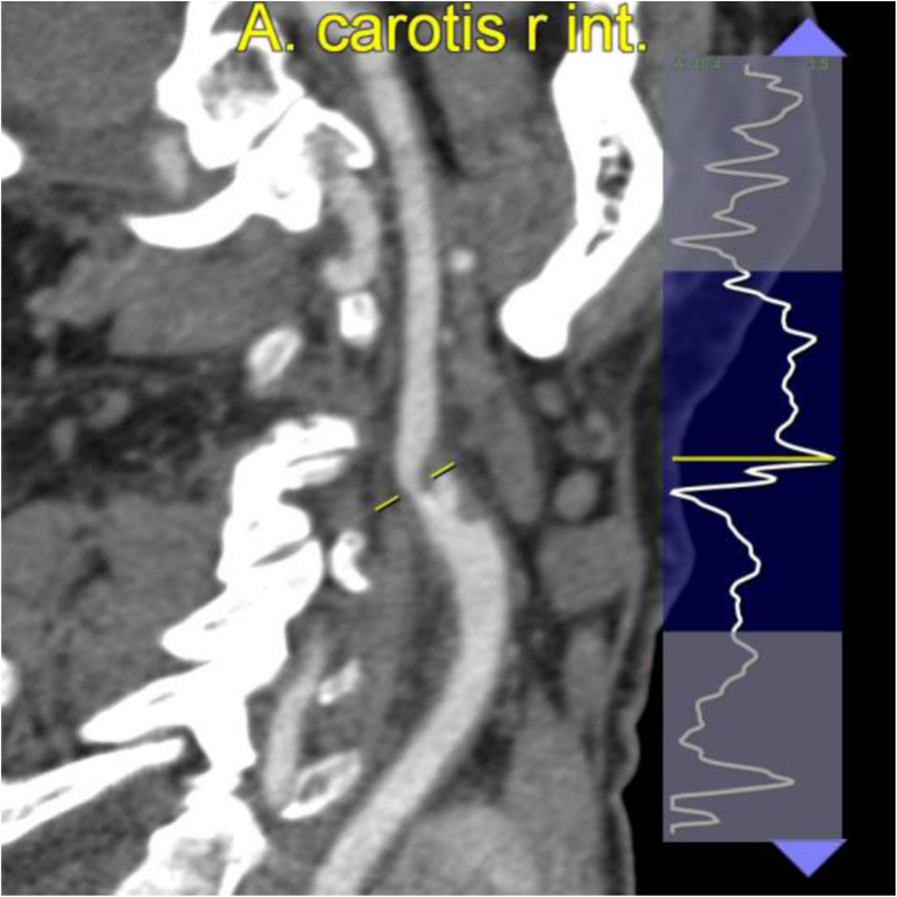
Cross-sectional sagittal carotid view. Severe carotid artery stenosis at the level of the right internal carotid artery, as indicated by the yellow line

**Fig. 2:**
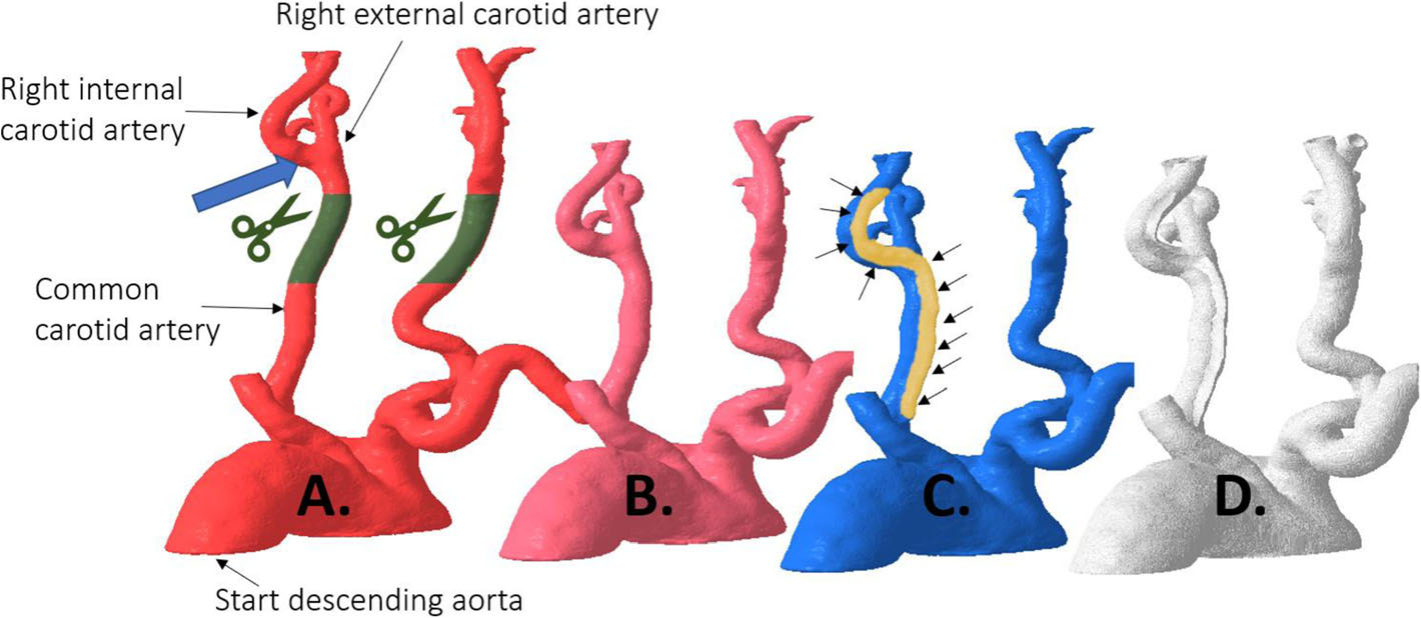
Posterior view of the aortic arch with subsequent editing steps to obtain the final model. **A**. In red, the anatomy is depicted as derived from the angio-CT. The part in green is cut out to truncate the model to fit within the building volume of the printer. The blue arrow indicates the stenosis location. **B**. The pink model is the result of the truncation at the common carotid artery level. **C**. The blue model shows the manually added and gold colored ridge, also indicated by the arrows. The golden ridge shows the position where the carotid artery will be deroofed. **D**. The white model shows the final deroofed model, which is the result of expanding the pink model B towards the outer edge by 1 mm and subtracting the blue model C. The white model is printed as depicted in Fig. 3

We present the case of an 80 year old male with severe stenosis in the right internal carotid artery, 0.5 cm cranial of the carotid bifurcation (Fig. 1). The bifurcation is situated 1.5 cm above the lower border of the mandibula. The preoperative CTA (0.75 mm slice thickness, 140kVp) was segmented and converted to a 3D printable .STL format using Mimics™ inPrint 3.0 software (Materialise, Leuven, Belgium). In order to print the model with correct internal vessel diameters in our limited desktop build volume, we chose to bilaterally shorten the common carotid arteries (from Fig. 2.A. to B.). We kept the aortic arch in place for a more realistic simulation, and applied a 1mm vessel wall thickness. To facilitate stent deployment view in the translucent material, we chose to deroof a viewing window at the level of the carotid bifurcation. The deroofing removes 25 % of the circumferential vessel wall and was achieved by modelling a ridge on the original vessel anatomy (Fig. 2.C.), which was subsequently subtracted from the 1 mm offset model resulting in our final model (Fig. 2.D.). Deroofing allowed for better visual feedback without requiring extra manual post-processing such as sanding or application of coatings which are frequently used to improve translucency after printing.

Truncation, fusion and ridge editing were performed in Meshmixer™ (Autodesk, California, USA), without omitting the internal vessel diameter. All arterial ends were left open. These open ends serve two goals. On one hand, they allow easy positioning and pull through of guidewires, allowing for a more realistic simulation. On the other hand, they facilitate internal flow of isopropyl alcohol which is used in post-processing to remove non-cured sticky resin.

**Fig. 3:**
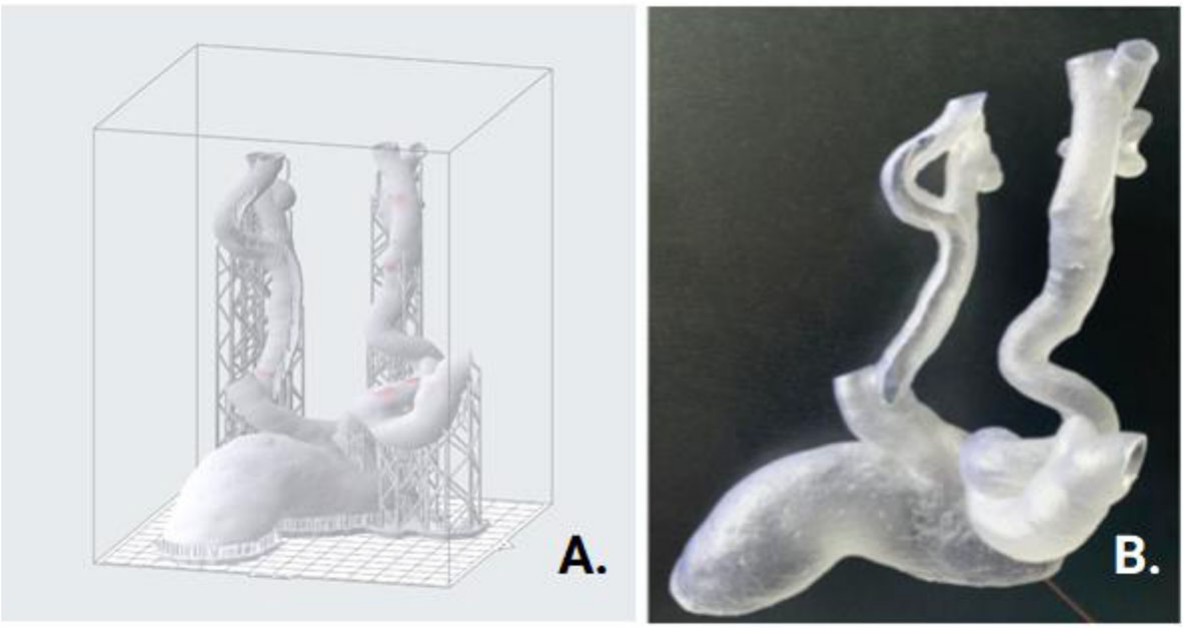
3D Printing Orientation and Final Model. A. Print build preparation as organized in PreForm™ (Formlabs, Somerville, Massachusetts, USA). Inside the carotid arteries, support strands are nearly completely avoided using this orientation. After printing, the model is cleaned with isopropyl alcohol and postcured using UV light in a Form Cure ™ module. Next, the outer strands are removed mechanically by hand. B. The 3D-printed model, with apparent translucency, printed in Clear Formlabs™ Resin. We note the wall of the right carotid artery is left open after deroofing. Catheter sheat is in place within the right internal carotid artery, ready for stent deployment

## Results

The model was printed in one single run on a desktop Formlabs™ 2 printer (Formlabs, Somerville, Massachusetts, USA) with build volume 145 × 145 × 175 mm. Total printing time attained 9h45min with a 0.1mm layer thickness. The model was oriented straight as to avoid internal support inside the vessels and was printed with 61.48 ml of standard Clear Formlabs™ Resin (Fig. 3).

**Fig. 4:**
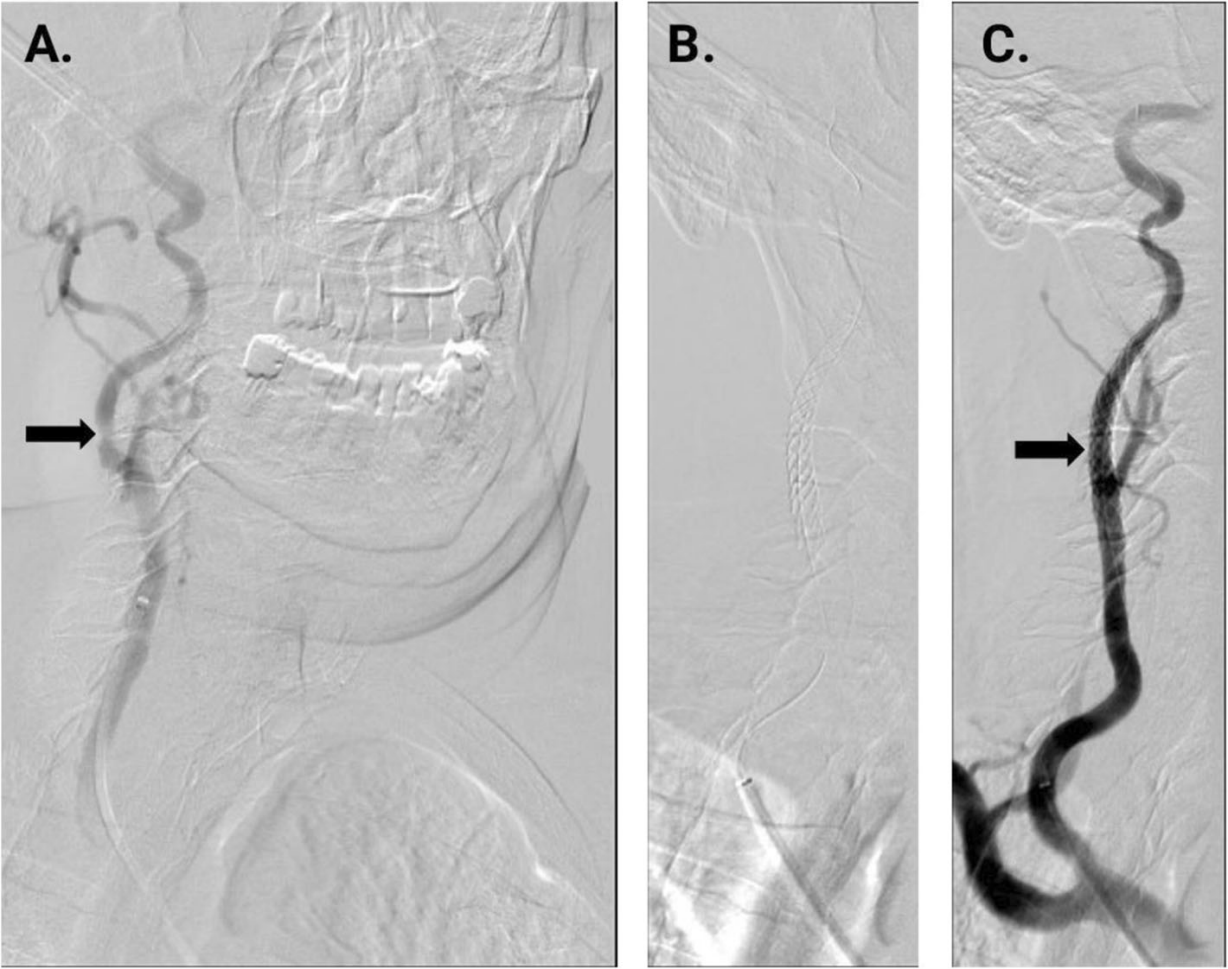
Stent deployment in vivo. **A**. High grade stenotic lesion in the right internal carotid artery, as indicated by the arrow. **B**. After placement of a Terumo™ Roadsaver stent (diameter 7mm, length 30mm). **C**. Angiography shows complete resolution of the stenosis after CAS without dissection of peripheral embolization

Figure 4 shows the in-patient stent positioning. A 90 cm 5Fr Terumo™ Destination Sheath (Terumo, Tokyo, Japan) and a 0.014 Terumo™ Gold DT wire was used in the live procedure. The lesion was stented using a Terumo™ Roadsaver stent (diameter 7mm, length 30mm). Post-dilatation was performed using a Boston Scientific™ Sterling balloon (diameter 6mm, length 20mm) after administration of 1 mg atropine.

**Fig. 5:**
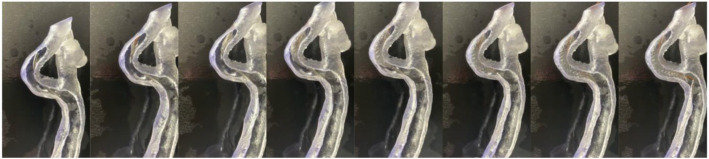
Stent deployment in silico. Serial series of stent deployment inside the internal carotid artery. The deroofed model allows for precise vision of the deployment, a feature which is not appreciable in completely closed translucent models. The stent is placed more distally to account for foreshortening. In real life, it would otherwise extend into the common carotid artery. We refer to the video in appendix to objectivate other features such as recoil and jumping and the end of deployment

Figure 5 shows the successful deployment of a date-expired 5 Fr Nitinol dual layer CAS stent inside the printed model. Deployment was performed without wire or sheat, as the model allowed for direct insertion of the stent delivery catheter inside the model. Deroofing allows for precise visual feedback of important features during CAS implantation. Foreshortening and release of the proximal stent end with accompanying recoil can readily be objectivated (videolink attached). Hence, this model provides unseen insights in stent positioning. It also allows for patient-specific procedure planning. The model was used by 5 experienced CAS surgeons during a workshop who described the tactile feedback as good as real life. This model was also used once in patient education and seemed to be very helpful. Comparison with post-procedure CTA was not performed due to poor renal function. As such, contrast enhanced follow-up imaging was avoided and luminal patency was assessed using duplex ultrasound only.

## Discussion

We demonstrate a proof-of-concept low-cost approach to teaching CAS. We specifically focus on objectivating deployment characteristics by direct vision, without fluoroscopy. A 3D printed model of the patient’s vascular anatomy including the ostial internal carotid stenosis could contribute to a proper risk factor assessment and surgical-approach analysis, as well as preoperative device sizing. These factors might in turn lead to preventing complications. We might even envision a possibility in extending the CAS indications through refined preoperative training and planning based on patient-specific 3D models. 3D printed models can enable training on difficult anatomical variations which cannot be observed in animal models. Given that CAS-procedure-specific-simulation can shorten the learning curve and lead to better patient outcomes [1], this approach can certainly be relevant in CAS training. In-hospital 3D-printing services can facilitate the introduction of these training models and give more residents access to training with limited costs. Deroofing of the model and accompanying editing steps, might be omitted by manual sanding and coating of the model after printing in an effort to obtain a transparent instead of translucent model. Although we visualize very specific deployment features thanks to our deroofing, this also might impact the stent-vessel interaction to a certain degree. It is however important to note that complete transparency might be hard to reach in UV cured polymers. These polymers tend to yellow out when exposed to daylight too long. As such, a deroofing approach is certainly relevant and avoids the use sanding and application of volatile coatings in a clinical environment.

The time constraint on 3D model development can be avoided by sharing the virtual ‘.STL’ or PreForm™ files between clinical training centers or co-development.

As this is a proof-of-concept study, it ignites many new opportunities for future research.

One drawback of our model is its rigidity, hence no tissue deformation can occur. Future tests should involve printing in the more recently developed elastic resins and investigate the impact of model deformation. Elastic models can be stretched open and real time flow pre -and post-treatment could then be simulated.

Although patient feedback on this model was positive, systematic patient-questionnaires with different 3D Printed carotid models should be conducted. Systematic trials could be set up to evaluate the effect on pre-operative planning, case-selection, stent-sizing or resident training. The focus of this model was on CAS deployment characteristics at the carotid level. Future studies can combine this local feature, with a larger model which also incorporates the descending and abdominal aorta. As such, pitfalls in guidance towards the carotid lesion as well as local pitfalls demonstrated here, can be addressed at once. It does need to be taken into account that, to our knowledge, this is not achievable in a cost-effective way or single print due to the small build volume of current desktop SLA printers. Nevertheless, flexible models for TCAR do not need the aortic arch to be printed as these procedures are completely performed at the carotid level. As such, flexible models that can be punctured and sutured, form an interesting research opportunity and alternative for these low-cost CAS training models.

## Conclusions

CAS has a very specific learning curve and currently, no 3D printed models exist to explain the pitfalls during the deployment phase without fluoroscopy. Preoperative patient-specific 3D printed models might enhance the patient’s outcome through case-specific training, accurate patient selection and preoperative planning. Patient education might also be improved using these models. Here, we present a framework to generate patient-specific 3D models in the point-of-care setting, which could allow for easier adaptation and implementation of these training models. However, more research is warranted to evaluate the value of these models.

## Supplementary Information

**Additional file 1.**

## Data Availability

Video-fragment available: https://drive.google.com/file/d/12bXLJJeazWQ8JsAhJCb8BQQAOmJQ4RUO/view?usp=sharing.

## Abbreviations

STL: Standard Tessellation Language
CAS: Carotid artery stenting
CEA: Carotid endarterectomy
CT: Computed tomography
2D: Two-dimensional
3D: Three-dimensional
CTA: Computed Tomography Angiogram
TCAR: TransCarotid Artery Revascularization

## Declarations

### Authors’ information

PDB is a 3th year urology resident with a master’s degree in biomedical engineering. CA is a 4th year medical student at Ghent University with a master degree in physiotherapy. CD is a professor in biomedical engineering (numerical biomechanics) at Ghent University. RB is a thoraco-vascular surgeon at OLV Hospitals.

## References

[1] Gosling AF, Kendrick DE, Kim AH, Nagavalli A, Kimball ES, Liu NT, et al. Simulation of carotid artery stenting reduces training procedure and fluoroscopy times. J Vasc Surg [Internet]. 2017;66(1):298–306. Available from: 10.1016/j.jvs.2016.11.066. 10.1016/j.jvs.2016.11.066 28533078

[2] Abbott AL, Paraskevas KI, Kakkos SK, Golledge J, Eckstein HH, Diaz-Sandoval LJ (2015). Systematic Review of Guidelines for the Management of Asymptomatic and Symptomatic Carotid Stenosis. Stroke.

[3] Kernan WN, Ovbiagele B, Black HR, Bravata DM, Chimowitz MI, Ezekowitz MD, et al. Guidelines for the prevention of stroke in patients with stroke and transient ischemic attack: A guideline for healthcare professionals from the American Heart Association/American Stroke Association. Stroke. 2014;Vol. 45:2160–236 p. 10.1161/STR.0000000000000024 24788967

[4] Bagley JH, Priest R. Carotid Revascularization: Current Practice and Future Directions. Semin Intervent Radiol. 2020 Jun;37(2):132–9. 10.1055/s-0040-1709154 PMC7224973 32419725

[5] Rosenfield K, Babb JD, Cates CU, Cowley MJ, Feldman T, Gallagher A, et al. Clinical competence statement on carotid stenting: Training and credentialing for carotid stenting - Multispecialty consensus recommendations: A report of the SCAI/SVMB/SVS Writing Committee to develop a clinical competence statement on carotid interventi. J Am Coll Cardiol [Internet]. 2005;45(1):165–74. Available from: 10.1016/j.jacc.2004.11.016. 10.1016/j.jacc.2004.11.016 15629399

[6] Ricotta JJ, Aburahma A, Ascher E, Eskandari M, Faries P, Lal BK. Updated Society for Vascular Surgery guidelines for management of extracranial carotid disease. J Vasc Surg [Internet]. 2011;54(3):e1–31. Available from: 10.1016/j.jvs.2011.07.031. 10.1016/j.jvs.2011.07.031 21889701

[7] Willaert WIM, Van Herzeele I (2013). Carotid artery stenting - strategies to improve procedural performance and reduce the learning curve. Interv Cardiol.

[8] Saleem T, Baril D. Carotid Artery Stenting. [Internet]. 2020. Available from: https://www.ncbi.nlm.nih.gov/books/NBK470541/. 29262170

[9] Lin PH, Bush RL, Peden EK, Zhou W, Guerrero M, Henao EA (2005). Carotid artery stenting with neuroprotection: Assessing the learning curve and treatment outcome. Am J Surg.

[10] Easton JD (2014). History of carotid endarterectomy then and now: Personal perspective. Stroke.

[11] Kachel R, Basche S, Schneider GH, Vogler E (1988). Die perkutane transluminale Angioplastik (PTA) von Hirngefäßstenosen (Indikation, Technik, Ergebnisse). Digitale bildgebende Verfahren Interventionelle Verfahren Integrierte digitale Radiologie.

[12] Müller MD, Lyrer PA, Brown MM, Bonati LH. Carotid Artery Stenting Versus Endarterectomy for Treatment of Carotid Artery Stenosis. Stroke. 2020;(January):E3–5. 10.1161/STROKEAHA.120.030521 33370200

[13] Cohen DJ, Stolker JM, Wang K, Magnuson EA, Clark WM, Demaerschalk BM (2011). Health-related quality of life after carotid stenting versus carotid endarterectomy: Results from CREST (Carotid Revascularization Endarterectomy versus Stenting Trial). J Am Coll Cardiol.

[14] Heck D, Jost A. Carotid stenosis, stroke, and carotid artery revascularization. Prog Cardiovasc Dis [Internet]. 2021;(xxxx):3–8. Available from: 10.1016/j.pcad.2021.03.005. 10.1016/j.pcad.2021.03.005 33744381

[15] Yang Y, Liu X, Xia Y, Wu W, Xiong H, Zhang H, et al. Impact of spatial characteristics in the left stenotic coronary artery on the hemodynamics and visualization of 3D replica models. Sci Rep [Internet]. 2017;7(1):1–13. Available from: 10.1038/s41598-017-15620-1. 10.1038/s41598-017-15620-1 PMC5684364 29133915

[16] Govsa F, Yagdi T, Ozer MA, Eraslan C, Alagoz AK (2017). Building 3D anatomical model of coiling of the internal carotid artery derived from CT angiographic data. Eur Arch oto-rhino-laryngology Off J Eur Fed Oto-Rhino-Laryngological Soc Affil with Ger Soc Oto-Rhino-Laryngology - Head Neck Surg.

[17] Petzold R, Zeilhofer HF, Kalender WA (1999). Rapid protyping technology in medicine–basics and applications. Comput Med imaging Graph Off J Comput Med Imaging Soc.

[18] Roguin A, Beyar R (2010). Real case virtual reality training prior to carotid artery stenting. Catheter Cardiovasc Interv.

[19] Nallamothu BK, Gurma HS, Ting HH, Goodney PP, Rogers M, Curtis M. JP, et al. Operator Experience Carotid Stenting Jama. 2011;306(12):1338–43. 10.1001/jama.2011.1357 PMC3208144 21954477

[20] Mas JL, Trinquart L, Leys D, Albucher JF, Rousseau H, Viguier A (2008). Endarterectomy Versus Angioplasty in Patients with Symptomatic Severe Carotid Stenosis (EVA-3S) trial: results up to 4 years from a randomised, multicentre trial. Lancet Neurol.

[21] Gray WA, Rosenfield KA, Jaff MR, Chaturvedi S, Peng L, Verta P. Influence of site and operator characteristics on carotid artery stent outcomes. JACC Cardiovasc Interv [Internet]. 2011;4(2):235–46. Available from: 10.1016/j.jcin.2010.10.009. 10.1016/j.jcin.2010.10.009 21349464

[22] de Vries EE, Kök M, Hoving AM, Slump CH, Toorop RJ, de Borst GJ (2020). In)comparability of Carotid Artery Stent Characteristics: A Systematic Review on Assessment and Comparison with Manufacturer Data. Cardiovasc Intervent Radiol.

[23] Willaert W, Aggarwal R, Bicknell C, Hamady M, Darzi A, Vermassen F, et al. Patient-specific simulation in carotid artery stenting. J Vasc Surg 2010;52(6):1700–5. Available from: 10.1016/j.jvs.2010.08.015. 10.1016/j.jvs.2010.08.015 20974522

[24] Göçer H, Durukan AB, Tunç O, Naseri E, Ercan E (2020). Evaluation of 3D printed carotid anatomical models in planning carotid artery stenting. Turkish J Thorac Cardiovasc Surg.

